# The road forward with swarm systems

**DOI:** 10.1098/rsta.2024.0135

**Published:** 2025-01-30

**Authors:** Hussein Abbass, Sanaz Mostaghim

**Affiliations:** ^1^ School of Systems and Computing, UNSW Canberra, Northcott Drive, Campbell, ACT 2600, Australia; ^2^ Faculty of Computer Science, Otto von Guericke University Magdeburg, Universitätsplatz, Magdeburg 39106, Germany

**Keywords:** artificial intelligence, swarm intelligence, swarm robotics, swarm systems, human–swarm teaming

## Abstract

Advances in artificial intelligence (AI) and robotics are accelerating progress in swarm systems. Large and bulky autonomous systems are being replaced with many, smaller, cheaper, distributed, decentralized and collectively smarter systems. However, developing these swarm intelligence systems comes with multiple challenges, including technological challenges to engineer smaller and smarter machines, interaction challenges to design novel interfaces and modalities for communication and sociotechnical challenges related to trustworthiness, ethics and legalities. Swarm systems present philosophical and mathematical dilemmas that are worthy of deeper scientific inquiry. This introduction contextualizes the topic in the contemporary literature and shows the current research directions and evolution of swarm systems using 11 carefully selected papers in this special theme. We conclude by discussing the way forward for swarm systems.

This article is part of the theme issue ‘The road forward with swarm systems’.

## 1. Introduction

Swarm systems have classically been defined as [1] ‘collections of autonomous, non-synchronized, non-intelligent robots cooperating to achieve global tasks’. However, they have evolved to the point that two components of this definition are no longer relevant. The first relates to the term ‘non-synchronized’. According to [2]

The term *synchronization* is used to refer to the solution of either of two distinct but related problems: (1) specification and control of the joint activity of cooperating sequential processes, or (2) serialization of concurrent access to shared objects by multiple processes.

The synchronization of shared objects in a decentralized system creates a bottleneck, meaning that it was avoided by historical swarm intelligence researchers [1]. However, the synchronization of computer systems is fundamentally different from that seen in nature. For example, when birds fly in a synchronized manner, it is as if they are all connected to a central computer. In this case, it may be assumed that the shared object is space—when one bird moves, it causes another to move. If the second bird resists movement, the first will avoid colliding with it by slowing down or changing direction. However, while synchronization is essential for self-organized behaviours in nature and emerges through an implicit sense–decide–act cycle, synchronization in classic computer systems relies on explicit communication.

The second issue relates to the word ‘non-intelligent’, which is problematic on many levels. While a bird may not be as intelligent as a human, this does not mean it is ‘unintelligent’. Perhaps the most problematic aspect of assuming that an agent in a swarm is ‘non-intelligent’ is the question of ‘why not?’ In other words, is it not possible for a swarm of ‘intelligent’, self-organizing machines to display a complexity that exceeds their individual capacities? Modern swarm systems comprise many smart machines, as seen in emerging research on the ‘thinking swarm’ [3].

Clearly, classical definitions of swarm systems are not relevant to modern-day swarm systems, calling for updated definitions. The following definitions establish a baseline for the contemporary literature on swarm systems:

**Definition 1.1 (individual agents).** *The smallest viable autonomous units in a system.*

**Definition 1.2 (collection of agents).** *A set of individuals.*

**Definition 1.3 (group of agents).** *A collection of agents with a shared identity (category).*

**Definition 1.4 (team of agents).** *A group of agents with shared objectives and goals.*

**Definition 1.5 (swarm of agents).** *A self-synchronized, efficient, effective team of agents.*

**Definition 1.6 (analytics).** *The assignment of meaning to measures.*

**Definition 1.7 (swarm analytics).** *Interpretation and explanation of swarm systems through analytics.*

These definitions allow a more sophisticated view of swarm systems. The definition of a swarm as an efficient and effective team reveals its basic characteristics: autonomous units that are grouped together according to their relationships and shared goals and objectives. While it may be debatable whether birds have shared goals and objectives when flocking together, we argue that goals and objectives do not always need to be consciously defined and assessed—in nature, they are genetically encoded. For example, birds avoid collisions to reduce the likelihood of injury and death. Therefore, agents can have objectives of which they are not even aware.

The above definition of swarm systems does not impose decentralization as a necessary or sufficient condition. In other words, a swarm may be either decentralized or centrally guided. For example, a sheepdog is a centralized means of guiding a swarm of sheep. In computer systems, swarms can be directed by a centralized control unit, such as those used in light shows [4]. Ideally, however, swarms are decentralized and rely on little to no explicit communications.

Despite the long history of swarm intelligence research [5], studies have been either theoretical or based on laboratory experiments. The lack of research on swarm systems in practice is only just emerging, with applications in the medical [6] and agricultural [7] domains.

The classical view of a swarm as a large collection of simple reactive entities has dramatically shifted in recent years with advances in artificial intelligence (AI), sensors, microchips and quantum-like technologies. We are currently on the verge of a fifth AI revolution in which intelligence is the product of not an artificial machine but a distributed network comprising both humans and swarms working collaboratively to achieve outcomes that are greater than those achievable by the individual parts. The nodes in these distributed networks consist of smarter swarms. This will have an even greater effect on humanity compared with large language models or human–AI augmentation.

Such a revolution begs the following questions: What is the current state of the art in swarm systems? What are the emerging conceptual and technological trends? What is the way forward in swarms? The 11 studies presented in this special theme attempt to answer these questions.

## 2. Theme summary

The 11 papers presented in this special theme issue can be clustered into three groups: those focused on the science of swarm systems; those focused on the technology of swarm systems; and those focused on the social integration of swarm systems, including cognitive engineering and ethical implications.

### (a) Swarm systems—scientific lens

Historically, swarms have been viewed as simple reactive systems. The global behaviour of swarms is the result of nonlinear local interactions and cannot be explained in terms of constituent parts. For example, while 20 is the product of four and five, it may also be the product of an infinite number of possibilities. Therefore, if the number 20 appears in a system, an observer who has no access to the individual agents cannot guarantee that they produced four and five. The flocking of birds is the result of not individual but collective actions. The phenomenon of nonlinearity in complex adaptive systems generally and swarm systems specifically has long fascinated scientists.

A reliance on simple rules and unexplainable but powerful emergent phenomena is challenging in classical engineering design, where the whole is divided into rational parts, each with a guaranteed performance, and the action of the whole can be explained by the inputs. This enables an explanation of system behaviour under normal operations and when something goes wrong. It is also challenging in cognitive multi-agent systems, which comprise rational agents. In economics, rationality is an optimization problem involving the maximization of the utility function of individual agents. This creates a dilemma when the utility of the group conflicts with the utility of individuals. Examples of these dilemmas in game theory include the prisoner’s dilemma, divide the dollar and the public goods game, which require strategies to engineer the choices of rational agents to achieve the best outcomes for the group.

Two critical questions related to swarm intelligence are: Can individuals in a swarm be rational agents, where nonlinear system-level behavioural outcomes still emerge in these systems? Can behavioural outcomes be explained in terms of individual agent utility? These questions are game changers because they create a bridge between the classical notion of a swarm, in which encoded reactions are based on sensory information, and the cognitive swarm, in which agents are rational players. While the former is simple and rapid, the behavioural outcomes of the group can be unpredictable. The latter is more complex, slow and deliberate, but the group’s behaviour can be engineered. By bridging these two ends of the spectrum, a swarm can change from unpredictable to predictable and utility agonistic to utility driven and adjust its speed depending on the context. In the paper ‘Swarms can be rational’, Gal Kaminka used game theory to model a swarm in which individuals are rational while collectively achieving a desired phenomenon [8].

The trade-off between swarm-level behavioural stability and the diversity of these behaviours has been a long-standing issue in the research on complex adaptive systems, artificial life and swarm intelligence. For example, if birds were to fly in the same formation indefinitely, the system would never produce anything new; rather, it would be more interesting to see them producing an infinite number of formations. In nature, this problem is addressed by open-ended evolution, in which evolution is unrestricted rather than bounded, producing a rich diversity of possibilities. For example, humans may eventually evolve to become cyborgs. While the problem is theoretically challenging for researchers of complex adaptive systems, it has significant practical implications when optimizing systems in dynamic environments. Hiroki Sayama, who has conducted groundbreaking work on swarm chemistry, has explored the use of swarm systems for creating open-ended evolutionary dynamics [9]. Swarm chemistry may be seen as mixing Reynolds [10] ‘boids’ model with category theory, where Sayama created boid groups with different profiles and demonstrated heterogeneous swarms on a large scale.

### (b) Swarm systems—technological lens

As a multidisciplinary technology, swarm systems are systems of systems. Most of the engineering and information science literature has contributed to the technological development of swarm systems; thus, it is difficult to imagine a way forward. However, swarm systems also unify components of this diverse set of disciplines. What is most important is the difference in inputs between swarm systems and general systems. Therefore, we categorize the technological lens into two categories: swarm analytics and swarm autonomy. Swarm analytics involves giving meaning to measures and enables agents to interpret the world, act, diagnose their actions and predict the future. Swarm autonomy transforms analytics into actions and behaviours by assessing the situation and risks, designing a course of action, selecting the appropriate action, controlling the actuators to transform planned actions into actual actions, directing sensors to receive appropriate feedback and using feedback from analytics to adapt future actions and processes. More categories can be added; for example, neither analytics nor autonomy considers the materials of swarm agents, sensor technologies or processors. However, in this article, we limit our discussion to swarm analytics and autonomy.

The area of swarm analytics [11] is relatively new. Jain *et al*. explored hub-based swarms, which consist of multiple agents that share a location-based hub (e.g. an ant nest) or an agent-based hub (e.g. a sheepdog) [12]. Because an information vector is needed to classify swarm states, assigning a meaning to each measure could result in an excessively long information vector. Autoencoders are machine learning models that reduce the dimensionality of an input vector by projecting it on to a significantly lower dimensional space known as the latent space. The encoding layer projects the input vector on to the latent space, while the decoding layer reconstructs the input vector from the latent space, maintaining the key data. In this way, any noise is filtered out of the data prior to classification. Equally, missing information in input vectors could be recovered by trained autoencoders.

Another example of a hub-based swarm can be seen in the case of a sheepdog guiding a swarm of sheep towards a goal. In classical shepherding, artificial sheepdogs must calculate the central mass of the sheep, which is normally done using the location of the sheep. Would the absence of an exact location make this guidance strategy ineffective? Li *et al*. [13] examined a case of shepherding when the sheepdog had limited information, showing that bearing information was sufficient for the sheepdog to successfully guide the swarm, even with intermittent communication and small measurement errors.

To work collectively, individuals in swarm systems share knowledge about the world through two primary means. The first is sensing, where passive or active sensors are used to collect information such as the distance to an obstacle or another agent. The second is communication, where messages move from a sending agent to a receiving agent. Direct communication relies on the explicit exchange of messages from one agent to another, while indirect communication involves a mediator that transmits the message from one agent to another. For example, ants communicate with each other indirectly through pheromones. The primary purpose of sensing and communication is for an agent to improve its decision-making processes; in nature, we assume the primary aim is survival. Cazenille *et al*. developed a taxonomy of the communication literature based on the compression of information and physical abstraction [14].

Most work on swarm systems has relied on heuristics, fixed scripts to control agents or machine learning models trained on experimental data. The control of swarm systems is challenging. However, the more we design models that are data-driven, the higher our understanding of the problem space, leading us to developing greater mathematical rigour. An example of a navigational challenge is when a swarm must navigate a constrained space [15]. The formation and topological structure of the swarm constrain its movements, causing disconnections between swarm members. Papaioannou *et al*. investigated a team-based version of this problem, where they formulated the problem as a distributed model predictive control problem [16].

Without quantum computing, swarm systems will not be ready for the future. While the world is yet to see a true quantum computer working at a large scale, quantum-like computers are emerging and working at scale. Apart from the emerging literature on quantum particle swarm optimization, research on quantum computing has neglected swarm systems. Mannone *et al*. review this emerging literature and focus their study on plausible quantum formulation of the rules governing swarm dynamics [17].

Previously, we mentioned the challenge of developing a classical control algorithm for swarm systems. It is equally difficult for swarm controllers to learn or search for possibilities without prior information. This challenge is being overcome using a machine education or teaching approach in which the problem is decomposed into parts. A curriculum to generate lessons for each part is designed to generate data. Machine learning models are designed for each part and the data generated from the corresponding curriculum are used to train these models. The resultant individual machine learning models are aggregated to obtain an overall solution for the control problem. Hussein *et al*. used two levels of autonomy and human demonstrations to generate data for machine learning models [18]. They showed that when swarms have higher levels of autonomy, humans are more efficient than teleoperated robots.

### (c) Swarm systems—socio-technical lens

The social integration of swarms brings multiple cognitive challenges because humans are not used to simultaneously managing multiple entities. For example, even with their extensive training, air traffic controllers can only manage approximately a dozen aircraft at any point in time while abiding by strict rules. This involves an increase in cognitive load and a decrease in situational awareness, which are further escalated by selective attention and bounded rationality. Social challenges are narrowly focused in social media with few discussions on agriculture, medicine or other civilian applications. Swarming also raises significant ethical and governance challenges.

Nalepka *et al*. used human-inspired strategies to encode autonomous swarms [19]. Lyons *et al*. also used human data to shed light on trust issues in human–swarm interactions, calling for more efficient interfaces for human–swarm interactions [20].

Ethics are challenging to consider when a technology makes decisions based on mathematical rather than human logic. Winfield *et al*. explore in their paper the ethics and governance of designing and operating swarm systems [21].

The next section offers suggestions for swarm systems based on the papers discussed above.

## 3. The way forward with swarm systems

### (a) Swarm systems—scientific lens

In terms of the science of swarm systems, Kaminka [8] and Sayama [9] offer initial steps in a complex area. We pose many questions that future researchers may explore: What does rationality mean in swarm systems? How should the utility function of each agent be designed so that interesting, self-organized behaviours emerge? How can individual-level utility function be linked to group-level utility function? Can the outcomes be influenced by engineering a hierarchy of utility functions in a swarm? How can a swarm that explores novel areas and displays richer behaviours indefinitely be designed? What is the relationship between the richness of the environment and the richness of the behaviours displayed by agents in a system exhibiting open-ended evolutionary capabilities?

### (b) Swarm systems—technological lens

In terms of the technology of swarm systems, the reviewed papers reveal numerous possible research directions and opportunities. The field of swarm analytics is only just emerging and needs development in terms of methodology, analytical frameworks and metrics. Hub-based approaches, whether location or agent based, are more suited to real-world applications. This concept is scalable to swarm hierarchies. The research in this area is in its infancy, with many questions that require deeper investigation: How can a scalable swarm hierarchy be designed? How can controllers for hub-based swarms be dynamically taught? What is the effect of latency in communication, noise, partial isolation or interruptions at the hubs?

More research is required at the interface of quantum computing and swarm systems. This theme only considered the limited problem of quantum computing for swarm systems. In addition to benefiting from quantum computing for optimization and machine learning models, swarm systems would benefit from quantum sensing and quantum communication. For example, localization is a challenge for mobile agents. Quantum positioning, navigation and timing could assist a swarm with localization and explicit time synchronization across the swarm network.

Machine education and machine teaching are emerging areas of machine learning. As more advanced autonomous machine learning models are developed, shifting machine learning from designing algorithms to designing systems capable of autonomous learning, the focus will shift more to frameworks and methodologies to educate and teach these intelligent agents. What pedagogical approaches are available for educating machines? How is machine education different from human education? What algorithms can better support machine education and machine teaching?

### (c) Swarm systems—socio-technical lens

In terms of the sociotechnical lens, the social integration of swarm systems is possibly one of the most difficult challenges of this century. While cognitive engineering studies into human–swarm interactions exist, most rely on simulated swarm experiments with simple levels of autonomy. It is unclear whether these findings are transferable to more complex levels of autonomy. While Hussein *et al*. [18] show that humans are more effective when swarms have a higher level of autonomy, how high is high? Are explainable and understandable swarms similar to explainable and understandable AI? What is a fully autonomous swarm system? What type of information cues should be used in the interface so that humans can understand and potentially work with swarms? This line of questioning naturally leads to ethical questions: Which ethical frameworks are appropriate for swarms? What does an ethically guided design for a swarm system look like, and does it differ from single-agent AI systems?

## Data Availability

This article has no additional data.
